# Pharmacological Modulation of the Cytosolic Oscillator Affects Glioblastoma Cell Biology

**DOI:** 10.1007/s10571-024-01485-2

**Published:** 2024-06-22

**Authors:** Paula M. Wagner, Santiago J. Fornasier, Mario E. Guido

**Affiliations:** 1 Centro de Investigaciones en Química Biológica de Córdoba (CIQUIBIC)-CONICET, Universidad Nacional de Córdoba, Córdoba, Argentina; 2 Departamento de Química Biológica Ranwel Caputto, Facultad de Ciencias Químicas, Universidad Nacional de Córdoba, Córdoba, Argentina

**Keywords:** Circadian rhythm, Glioblastoma, Chronotherapy, Metabolic oscillator

## Abstract

The circadian system is a conserved time-keeping machinery that regulates a wide range of processes such as sleep/wake, feeding/fasting, and activity/rest cycles to coordinate behavior and physiology. Circadian disruption can be a contributing factor in the development of metabolic diseases, inflammatory disorders, and higher risk of cancer. Glioblastoma (GBM) is a highly aggressive grade 4 brain tumor that is resistant to conventional therapies and has a poor prognosis after diagnosis, with a median survival of only 12–15 months. GBM cells kept in culture were shown to contain a functional circadian oscillator. In seeking more efficient therapies with lower side effects, we evaluated the pharmacological modulation of the circadian clock by targeting the cytosolic kinases glycogen synthase kinase-3 (GSK-3) and casein kinase 1 ε/δ (CK1ε/δ) with specific inhibitors (CHIR99021 and PF670462, respectively), the cryptochrome protein stabilizer (KL001), or circadian disruption after *Per*2 knockdown expression in GBM-derived cells. CHIR99021-treated cells had a significant effect on cell viability, clock protein expression, migration, and cell cycle distribution. Moreover, cultures exhibited higher levels of reactive oxygen species and alterations in lipid droplet content after GSK-3 inhibition compared to control cells. The combined treatment of CHIR99021 with temozolomide was found to improve the effect on cell viability compared to temozolomide therapy alone. *Per*2 disruption affected both GBM migration and cell cycle progression. Overall, our results suggest that pharmacological modulation or molecular clock disruption severely affects GBM cell biology.

**Supplementary Information:**

The online version contains supplementary material available at 10.1007/s10571-024-01485-2.

## Introduction

The circadian system in all vertebrates, including mammals, temporally regulates behavioral, physiological, and molecular rhythms with a period close to 24 h, allowing organisms to anticipate the environmental cycles with implications for health and disease (Dunlap et al. 2004). The system comprises the central clock in the suprachiasmatic nucleus (SCN) of the hypothalamus and peripheral clocks in different tissues throughout the body. The central clock receives light, the main synchronizer of the circadian system, through intrinsically photosensitive retinal ganglion cells and is thus able to coordinate physiology and behavior through outputs sent to the peripheral clocks. These circadian oscillators are present even in individual cells and constitute a highly conserved molecular machinery in diverse species (Bass and Takahashi 2010; Golombek and Rosenstein 2010; Guido et al. 2010).

At the molecular level, the circadian machinery involves transcriptional–translational feedback loops (TTFLs) responsible for generating the ~24-h oscillations in gene expression. In the main loop, the positive elements of the circuit, BMAL1, and CLOCK, heterodimerize and recognize E-box sequences in the promoter of the period (*Per*1, *Per*2, *Per*3) and cryptochrome (*Cry*1, *Cry*2) genes to activate their transcription. Once the negative regulators PER and CRY accumulate in the cytoplasm and heterodimerize, they translocate to the nucleus to repress their own transcription by interacting with the BMAL1: CLOCK complex. Degradation of the negative regulators also plays a role in closing a 24-h cycle and starting a new cycle. The secondary loop includes the nuclear receptors REV-ERB α/β and ROR α/β/γ, which recognize retinoic acid-related orphan receptor response elements (RORE) in the promotor region of target genes. In turn, REV-ERB proteins repress *Bmal*1 and *Npas*2 transcription whereas ROR proteins can activate *Clock* and *Bma1*1 expression (reviewed in (Guido et al. 2022). In light of recent evidence of PER2 protein oscillation in a CRY-deficient model, Putker and colleagues (2021) propose that the TTFL is required for the robustness of behavioral and physiological rhythmicity but is dispensable for circadian timekeeping per se. This suggests the existence of an alternative coupled underlying timekeeping mechanism termed “cytoscillator” involving the casein kinase 1 δ/ϵ (CK1δ/ϵ) and glycogen synthase kinase 3 (GSK-3) (Putker et al. 2021). This post-translational mechanism is evolutionarily conserved across a range of model systems including isolated red blood cells (reviewed in (Wong and O’neill 2018; Li et al. 2023)) and can function independently of canonical clock proteins (Qin et al. 2015). Selective pharmacological inhibitors for the cytosolic kinases CK1δ/ϵ and GSK-3 such as PF670462 and CHIR99021, respectively, have been previously shown to regulate the speed at which the cellular clock works in several model organisms (Badura et al. 2007; Hirota et al. 2008; Causton et al. 2015). Also, the small molecule KL001, a ubiquitin-dependent degradation inhibitor of CRY has been shown to length the circadian period (Hirota et al. 2012).

In particular, GSK-3 is a serine/threonine protein kinase with two functional isoforms, α and β. The latter isoform is a protein kinase of 47 kDa that regulates cellular processes such as proliferation, cell cycle control, migration, invasion, and apoptosis (reviewed in (Sahin et al. 2019)). Evidence in the literature suggests that GSK-3 phosphorylates at least five clock proteins such as PER2, CRY2, CLOCK, BMAL1, and REVERBα (Sahar et al. 2020; Kaladchibachi et al. 2007; Spengler et al. 2009; Kurabayashi et al. 2010). However, GSK-3-mediated phosphorylation could have differential effects on the stability of the targeted substrates; BMAL1 phosphorylation by GSK-3 leads to ubiquitination and degradation by the proteasome whereas this post-translational modification in REV-ERBα protein protects it from degradation (Besing et al. 2015).

Although circadian disruption could alter metabolic pathways that lead to pathologies, little is known about the pharmacological modulation of the cytosolic kinases as a potential therapeutic strategy to treat brain cancer. Based on the World Health Organization (WHO) classification, glioblastomas (GBMs) are grade 4 brain tumors resistant to conventional therapies; they represent 45.2% of all malignant central nervous system (CNS) tumors and 80% of all primary malignant CNS tumors (Thakkar et al. 2014). The standard of care treatment is the Stupp protocol, which combines surgery and radiotherapy followed by the administration of the DNA-alkylating chemotherapeutic temozolomide (TMZ) (reviewed in (Wagner et al. 2021a)). Though the TMZ regimen was approved by the US Food and Drug Administration (FDA), its chemotherapeutic efficiency is poor, and most patients relapse (Stupp et al. 2009). Previously, we reported that T98G cells derived from a human GBM contain a functional clock that regulates metabolic oscillations and temporal susceptibility to Bortezomib administration (proteasome inhibitor) (Wagner et al. 2018). In vivo studies in our laboratory demonstrated higher efficacy in impairing tumor growth when Bortezomib was applied at night to tumor-bearing mice compared to diurnal administration, even at lower drug dosage (Wagner et al. 2021c). Moreover, T98G cultures showed significant differences in cell viability across time when cells were treated with SR9009 (synthetic REV-ERB agonist). This effect was further potentiated when SR9009 was combined with Bortezomib (Wagner et al. 2019).

Regarding the expression of GSK-3 in brain tumors, Miyashita and collaborators reported higher expression levels and activity of GSK-3β in GBM compared with normal brain tissues (Miyashita et al. 2009). Even though GSK-3β has been postulated as a tumor suppressor in some types of cancer, this kinase can serve as a pro-oncogene in other malignancies such as pancreas, colorectal, hepatocellular carcinoma, kidney, leukemia, and GBM (reviewed in (Sahin et al. 2019)).

It should be noted that environmental alterations, genetic mutations, or changes in circadian gene expression can disrupt the normal functioning of the circadian machinery and increase the risk of cancer occurrence and progression (reviewed in Sulli et al. 2019; Ortega-Campos et al. 2023)). The International Agency for Research on Cancer (IARC) classified “shift work leading to a circadian disruption” as a probable human carcinogen (Group 2A) (Stevens et al. 2011). In this sense, studies have shown that the negative element PER2 plays a critical role in GBM growth and metabolism (Yao et al. 2023). Moreover, PER protein expression is significantly downregulated in glioma tissues with respect to normal brain samples (Ma et al. 2020) (reviewed in (Wagner et al. 2021a)).

In brief, we hypothesize that the pharmacological modulation of the circadian machinery and the genetic disruption of the molecular clock could severely affect GBM biology. To test this hypothesis and to find novel strategies to treat GBM, we evaluated the effects of 1) synthetic inhibitors of cytosolic kinases such as CHIR99021 (GSK-3 inhibitor) and PF670462 (CK1ε/δ inhibitor), 2) the cryptochrome protein stabilizer (KL001), or 3) clock gene *Per*2 disruption on GBM T98G cell biology.

## Results

### Circadian Clock Modulation and Genetic Disruption Implications for GBM Cell Viability and Migration

#### Pharmacological Modulation: PF670462, CHIR99021, and KL001 Treatment

**Fig. 1:**
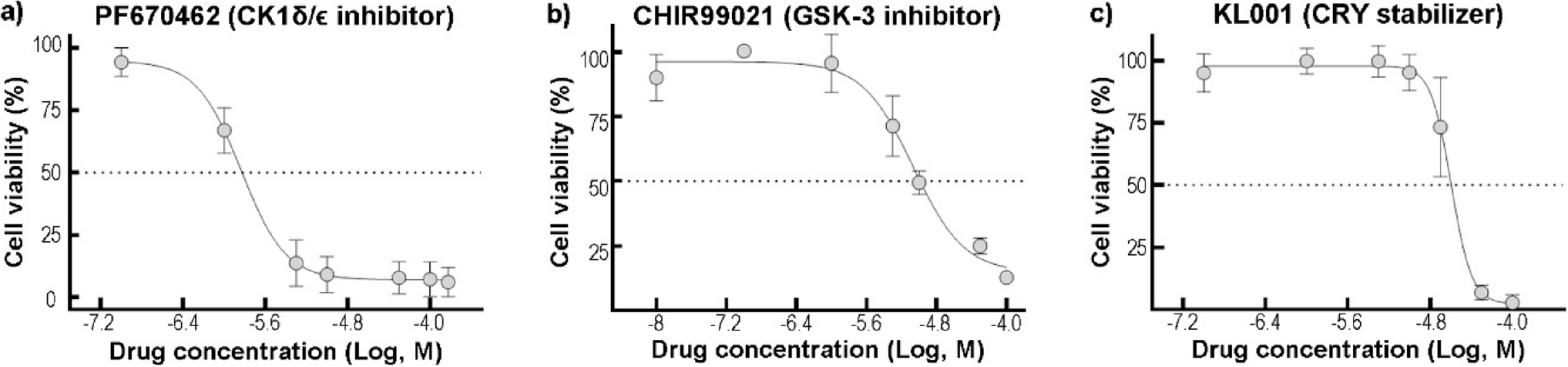
Effect of circadian pharmacological treatment on GBM cell viability. Dose–response curves of PF670462 (**a**), CHIR99021 (**b**), and KL001 (**c**) in T98G cells after 48 h of treatment showed an IC50 value of 1.5 μM (R^2^ = 0.96), 8.6 μM (R^2^ = 0.945), and 25.4 μM (R^2^ = 0.96), respectively. The results are mean ± SD of two independent experiments (n = 3/group)

**Fig. 2:**
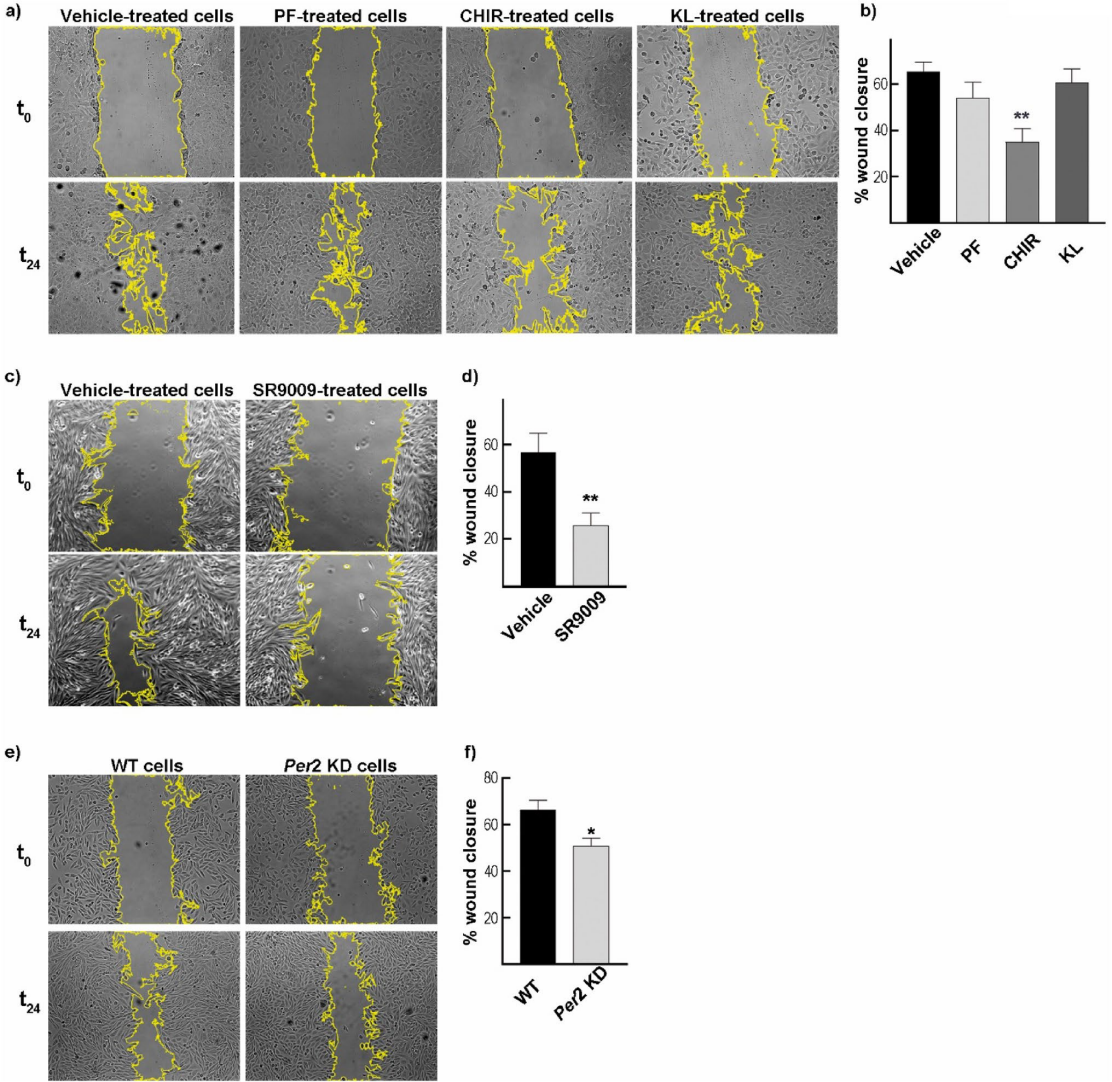
Effect of the pharmacological modulation and *Per*2 KD on GBM cell proliferation and migration. **a** Representative microphotographs of the scratch done on the monolayer of T98G cells incubated with DMSO (vehicle), PF670462 (1.5 μM), CHIR99021 (8.6 μM), or KL001 (25.4 μM) were taken at t_0_ and t_24._ **b** Wound closure percentage showed a significant decrease in the covered area of cells incubated with CHIR99021 as compared to vehicle-treated cells. No significant differences were observed between PF670462 and KL001 conditions compared to vehicle-treated cells. PF: PF67046; CHIR: CHIR99021; KL: KL001. **c** Representative microphotographs of the scratch on SR9009-treated (20 μM) and control cells were taken at t_0_ and t_24_. **d** Wound closure percentage showed a significant decrease in the covered area of SR9009-treated cells compared to vehicle-treated cells. **e** Representative microphotographs of the scratch done on the monolayer of *Per*2 KD and WT T98G cells were taken at t_0_ and t_24._ **f** Wound closure percentage showed a significant decrease in the covered area of *Per*2 KD cells as compared to WT cultures. The results are mean ± SEM of two/three independent experiments performed in triplicate. *p < 0.05, **p < 0.01

To investigate whether pharmacological modulation of the circadian clock affects GBM viability, T98G cells were treated with a CK1ε/δ inhibitor (PF670462), a GSK-3 inhibitor (CHIR99021), or a CRY protein stabilizer (KL001) in a range of concentrations from 0.01 to 100 μM for 48 h. The dose–response curve evidenced a half-maximal inhibitory concentration (IC50) of 1.5 μM (R^2^ = 0.96), 8.6 μM (R^2^ = 0.94), and 25.4 μM (R^2^ = 0.96) for PF670462, CHIR99021, and KL001, respectively (Fig. 1). The effect of GSK-3 inhibition on cell viability was also tested in another glioma cell line, showing an IC50 of 38.7 μM (R^2^ = 0.92) when murine GL26 cells were treated with CHIR99021 for 48 h. On the contrary, an IC50 of 91.2 μM (R^2^ = 0,99) was evidenced in the non-cancerous NIH 3T3 cell line (Suppl. Figure 1), a concentration ten times greater than that observed for T98G cells. The value of R^2^ denotes the goodness of fit of the experimental data with a symmetrical sigmoidal curve. The determined IC50 values were used to perform subsequent experiments. Wound-healing experiments were assayed after T98G cell treatment at the mentioned IC50 for 24 h to elucidate whether the pharmacological modulation impairs tumor proliferation and migration. Results showed that vehicle-treated cells had covered about 65.1% of the wound 24 h after the injury whereas cells incubated with the inhibitor CHIR99021 filled only 35% of the gap (p < 0.021 by unpaired t-test). On the contrary, no significant differences were observed in the percentage of wound closure between T98G cells treated with the CK1ε/δ inhibitor or the CRY protein stabilizer and control cultures (Fig. 2a,b). Interestingly, lower expression levels of vimentin, an intermediate filament protein implicated in migration and epithelial-mesenchymal transition of tumor cells, were observed in CHIR99021-treated cells (p < 0.0088 by unpaired t-test) (Suppl. Figure 3a,b). Since only GSK-3 inhibition evidenced significant effects on cell viability and wound healing experiments, we decided to use the compound CHIR99021 for the following experiments to investigate deeply its effects on GBM biology.

In another series of experiments, we evaluated the effect of SR9009 treatment on T98G migration. By acting as a REV-ERBs-specific agonist, this synthetic pyrrole derivative can modulate the circadian clock function. Previously, our laboratory demonstrated that SR9009 treatment decreased T98G cell viability and impaired cell cycle progression (Wagner et al. 2019). In this case, the percentage of wound closure of SR9009-treated cells (~26%) was significantly lower than that observed in cultures incubated with the vehicle (~57%) (p < 0.006 by Mann Whitney test) (Fig. 2c–d). Variations observed in cell morphology can be due to cell stocks coursing different replication ages. Nevertheless, similar results for cytotoxicity or wound healing experiments were obtained regardless of the stock utilized. Figures 2a and c were performed using the Leica DMI 8 and Axiovert 135 microscope, respectively.

#### Genetic Disruption: Per2 Knockdown Model

To determine if the molecular clock disruption affects GBM cell proliferation and migration, wound healing experiments were carried out in WT cells or *Per*2 knockdown (KD) cultures. The results indicated that cells with altered expression of PER2 filled only 51% of the gap compared with 66% of wound closure observed in WT cultures (p < 0.021 by Mann–Whitney test) (Fig. 2 e–f).

### Cell Cycle Distribution After CHIR99021 Treatment or *Per*2 Disruption

**Fig. 3:**
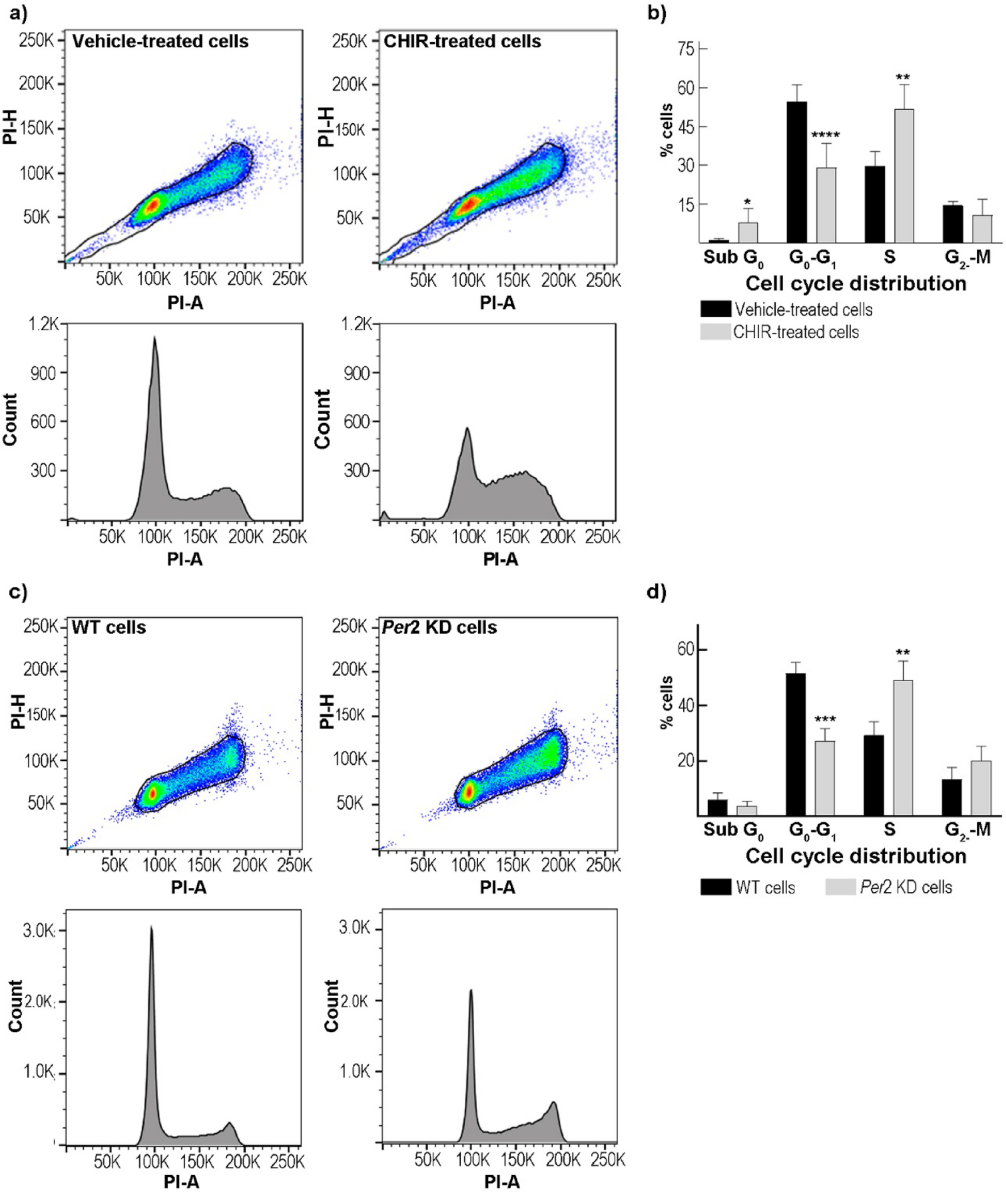
GSK-3 inhibitor treatment and *Per*2 downregulation effects on GBM cell cycle progression. **a** Representative cell cycle distribution of T98G cells incubated with CHIR99021 (8.6 μM, right column) or DMSO (vehicle, left column). **b** Histograms of the percentage of cells in each phase showed a higher proportion of CHIR99021-treated cells in the sub-G_0_ and S phase and a decrease in the G_0_–G_1_ phase compared to control cultures. No significant differences were observed in the proportion of cells in the G_2_-M phase between the experimental conditions. CHIR: CHIR99021. **c** Representative cell cycle distributions of WT (left column) and *Per*2 KD (right column) T98G cells arrested in serum-free DMEM and then stimulated with 20% FBS. **d** Quantification of cell cycle distribution showed a lower percentage of *Per*2 KD cells in the G_0_–G_1_ phase and a higher proportion in the S phase with respect to WT cultures. The results are mean ± SEM of three independent experiments performed in triplicate. *p < .0.05, **p < 0.01, ***p < 0.001, ****p < 0.0001 by unpaired t-test

To explore whether the cell viability and migration experiments could be linked to cell cycle distribution, T98G cells were treated with the GSK-3 inhibitor for 24 h, fixed, and stained with propidium iodide. Flow cytometry results showed a higher percentage of cells in the sub-G_0_ phase on CHIR99021-treated (~6%) cells compared to control cultures (~1%) (p < 0.01 by unpaired t-test). The percentage of cells in the G_0_-G_1_ phase among treated cells was significantly lower (~29%) than in vehicle-treated cells (~51%) (p < 0.0001 by unpaired t-test). In contrast, the S-phase showed a significantly higher proportion of cells in CHIR99021-treated cultures (~53%) than in control cells (~38%) (p < 0.0014 by unpaired t-test). No significant differences were observed in the G_2_-M phase among the experimental conditions (Fig. 3a, b). Since *Period* genes are downregulated in glioma samples compared to normal brains (Ma et al. 2020; Wagner et al. 2021b), we evaluated how the disruption of *Per*2 affects cell cycle progression. For this, WT and *Per*2 KD cells were arrested in FBS-free DMEM for 48 h and then stimulated with 20% FBS in DMEM medium allowing cultures to re-enter the cell cycle. Significant differences were observed in the G_0_-G_1_ phase with 52% and 27% of cells in WT and *Per*2-KD cultures, respectively (p < 0.0002 by unpaired t-test). By contrast, a higher percentage of cells were found in the S-phase (49%) after *Per*2 disruption than in the WT condition (29%) (p < 0.003 by unpaired t-test). However, no significant differences were found in the sub-G_0_ or G_2_/M phases between the experimental conditions examined (Fig. 3c–d). The results may reflect a greater proliferative proportion of *Per2* KD cells, likely due to cells retained in the S-phase.

### Characterization of Clock Gene Expression on GBM Cells After CHIR99021 Treatment or *Per*2 Disruption

**Fig. 4:**
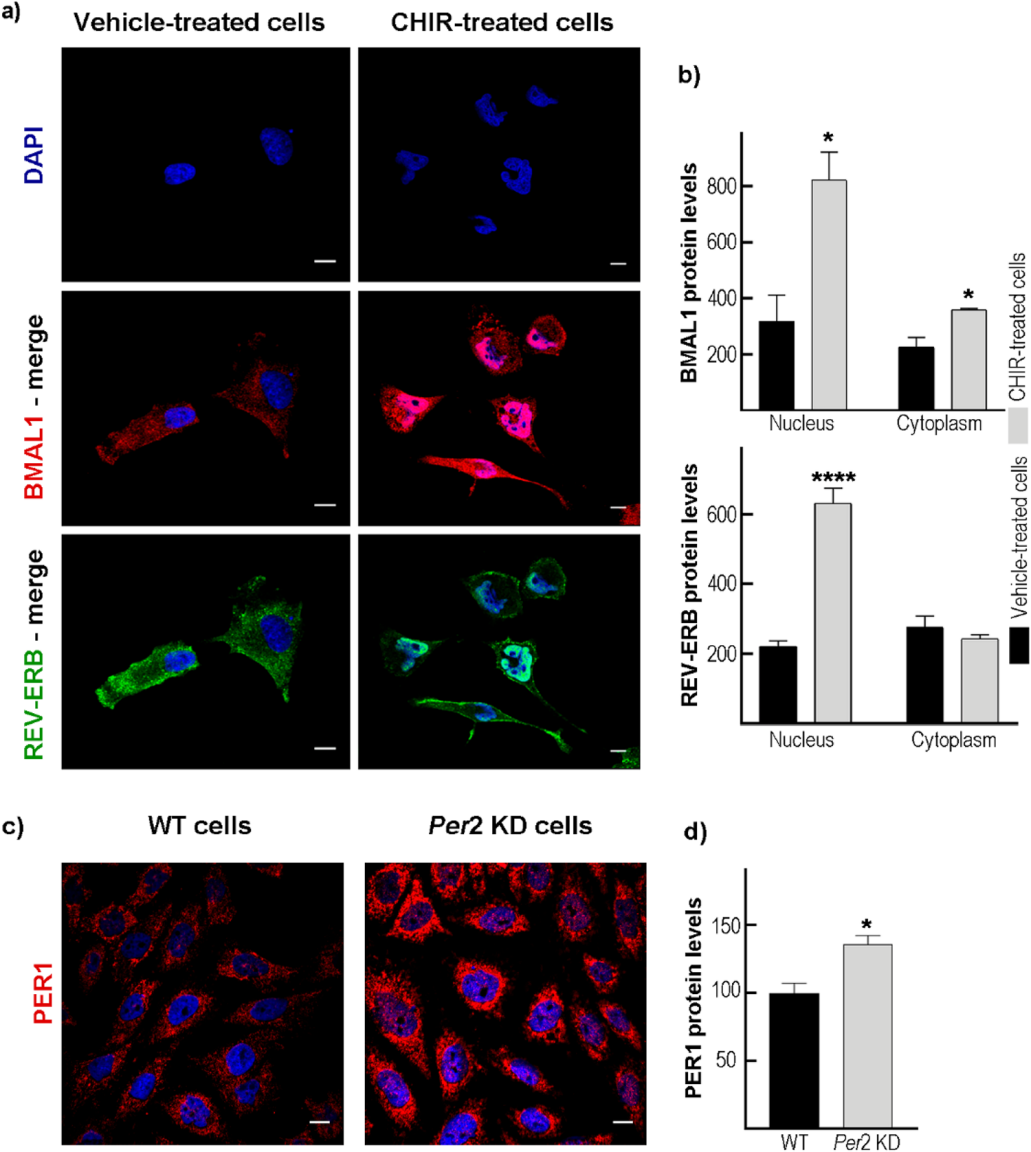
Characterization of clock gene expression after CHIR99021 treatment and *Per*2 KD on GBM cells. **a** Representative microphotographs of vehicle- (left column) or CHIR99021-treated cells (right column) immunolabeled with antibodies for BMAL1 (red), REV-ERBα (green), and DAPI for nuclear localization (blue). Scale bar = 10 μm. **b** Quantification of BMAL1 levels (upper graph) showed a higher expression both in the nucleus and cytoplasm in T98G cells treated with CHIR99021 (gray bars). In contrast, REV-ERBα expression (bottom graph) only showed higher levels in the nucleus as compared with control cells (black bars). CHIR: CHIR99021. **c** Representative microphotographs of WT (left column) and *Per*2 KD (right column) T98G cells immunolabeled with the antibody for PER1 (red), and DAPI for nuclear localization. Scale bar = 10 μm. **d** Quantification of PER1 protein levels showed higher expression in *Per*2 KD (gray bar) cells compared to WT cultures (black bar). *p < 0.05, ***p < 0.0001 by unpaired t-test

To further investigate the effect of pharmacological modulation on the circadian machinery, T98G cells were treated with the IC50 of CHIR99021 for 24 h, and clock protein expression was evaluated by immunocytochemistry. Results evidenced higher protein levels of the clock activator BMAL1 in cells treated with CHIR99021 compared to control cultures both in the nucleus (p < 0.028 by Mann–Whitney test) and in the cytoplasm (p < 0.0244 by unpaired t-test). On the contrary, levels of REV-ERBα protein showed a significantly higher expression only in the nucleus of CHIR99021-treated cells as compared with vehicle-incubated cells (p < 0.0001 by unpaired t-test) (Fig. 4a, b). Similar results were observed by western blot (Suppl. Figure 3a, b), which showed higher levels of BMAL1 protein in T98G-treated cells with the GSK-3 inhibitor. On the other hand, higher levels of PER1 protein were observed by immunocytochemistry in T98G cells after *Per*2 disruption with respect to WT cells (p < 0.0011 by unpaired t-test) (Fig. 4c–d), likely indicating a compensatory effect on *Per1* or the lack of the repressor activity after *Per2* KD. Additional effects were found on the expression of the clock activator *Bmal*1 by PCR (Suppl. Figure 2b).

### Metabolic Alterations After CHIR99021 Treatment

#### Redox State

**Fig. 5:**
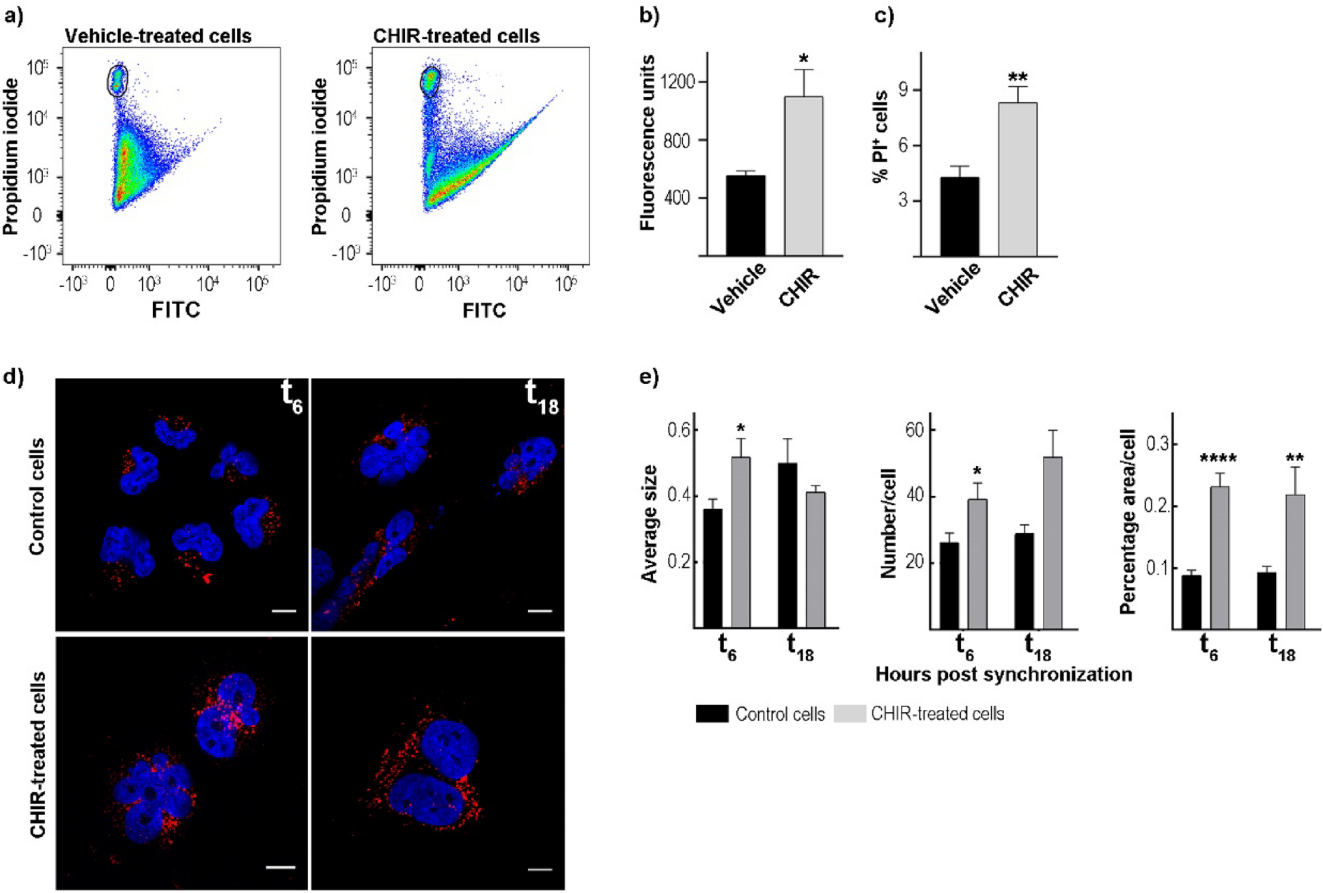
Redox state and lipid droplet determination in GBM cells after GSK-3 inhibition. **a** Representative dot plots of propidium iodide versus ROS levels (FITC) analyzed in CHIR99021 (8.6 μM) or vehicle-treated cells by flow cytometry. Quantification of fluorescence levels of ROS (**b**) and positive propidium iodide cells (**c**) showed higher levels in cells treated with the GSK-3 inhibitor (gray bars) compared to the control cultures (black bars). The results are mean ± SEM of three independent experiments performed in triplicate. *p < 0.05 by unpaired t-test, **p < 0.01 by Mann Whitney test. **d** Representative microphotographs of LD staining in T98G cultures or CHIR99021-treated cells synchronized by a 1-h pulse of DEX and fixed at 6 (left column) and 18 (right column) hours after synchronization. LD fluorescence staining of Bodipy is shown in red, and nuclei stained with DAPI in blue. Scale bar = 10 μm. **e** Quantification of LD parameters showed a significant increase in average size, percentage area, and number of LDs after GSK-3 inhibition (gray bar) when the cells were fixed at 6 h after synchronization as compared with control cultures (black bar); CHIR99021-treated cells only showed higher levels in terms of LD percentage area with respect to non-treated cells fixed at 18 h after DEX-pulse. *p < .0.05, **p < .0.001, ***p < .0.0001 by unpaired t-test or Mann–Whitney test. *PI* propidium iodide, *CHIR* CHIR99021

To investigate the ability of the GSK-3 inhibitor to alter the redox state, T98G cells were incubated with CHIR99021 at a final concentration of 8.6 μM for 24 h, and reactive oxygen species (ROS) levels were measured using the fluorescent probe 2′,7′-dichlorodihydrofluorescein diacetate. The results analyzed by flow cytometry showed a significant increase in ROS levels in CHIR99021-treated cells with respect to vehicle-treated cells (p < 0.014 by unpaired t-test) (Fig. 5a, b). Interestingly, a higher percentage of positive propidium iodide cells was observed in dot plots of flow cytometry in cells incubated with the GSK-3 inhibitor as compared to control cultures (p < 0.004 by Mann Whitney test), strongly indicating positive propidium iodide staining of dead cells (Fig. 5a–c).

#### Lipid Droplets

Considering that BMAL1 and REV-ERB are targets of post-translational modifications by the GSK-3, and these clock proteins are closely implicated in cellular metabolism, we evaluated whether the pharmacological modulation of the circadian machinery could alter the cytosolic/metabolic oscillator. We analyzed different parameters of lipid droplets (LDs), the cytoplasmic organelles mainly involved in energy storage (Farese and Walther 2009; Beller et al. 2010; Brasaemle and Wolins 2012). To this end, fixed cells were stained with Bodipy dye, which has highly lipophilic neutral characteristics that allow it to pass through the cell membrane to localize polar lipids and specifically stain LDs (Qiu and Simon 2016). Confocal microscopy results showed a larger average size and number of LDs in T98G cells fixed 6 h after dexamethasone (DEX) synchronization in CHIR99021-treated cells (0.52 μm^2^ and ~ 39 LDs/cell, respectively) as compared to control cultures (0.36 μm^2^ and ~ 26 LDs/cell, respectively) (p < 0.02 and p < 0.03 by unpaired t-test, respectively); no significant differences between the two experimental groups were observed at 18 h after synchronization. However, there was a significantly higher percentage area of LDs in T98G cells previously incubated with the GSK-3 inhibitor both at 6 and 18 h after DEX synchronization with respect to control cells (p < 0.0001 by unpaired t-test and p < 0.003 by Mann Whitney, respectively) (Fig. 5d–e). These results strongly indicate a significant effect of the GSK-3 inhibitor on LD levels at the times evaluated showing the highest impact at 6 h after synchronization compared to control cultures. The differences observed in LD parameters after GSK-3 inhibition could result from, at least in part, the tight regulation by the circadian clock as previously shown for the LD content (Monjes et al. 2022) and other metabolic parameters in different cancer cells (Wagner et al. 2018, 2019) (reviewed in (Guido et al. 2022)) as well as the differential response to pharmacological modulation of clock proteins.

### Temporal Susceptibility to Combined Treatment with Temozolomide and CHIR99021

**Fig. 6:**
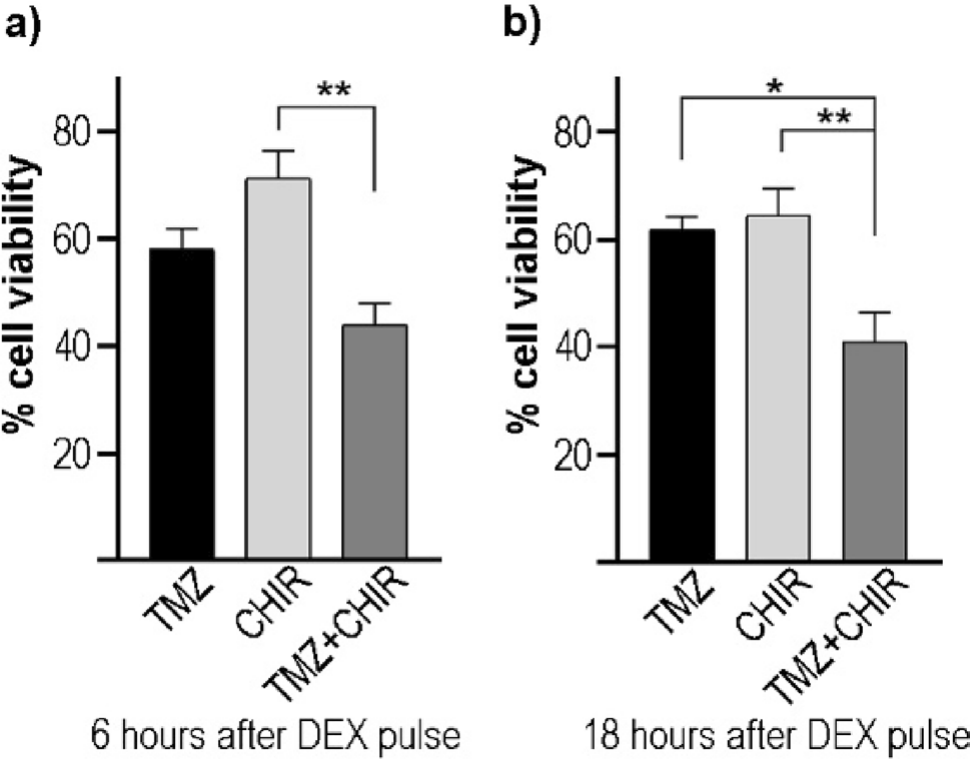
Temporal cell susceptibility to combined treatment with TMZ and CHIR99021. T98G cells were synchronized by a 1-h pulse of DEX and treated with the drugs at 6 and 18 h after synchronization for 48 h. Cell viability results revealed a significant effect when cells were treated with TMZ (844 μM) and CHIR99021 (8.6 μM) at 18 h after synchronization compared to treatment with each drug alone (**b**). On the contrary, the combination of drugs at 6 h after DEX synchronization showed a significant reduction in cell viability compared to CHIR99021-treated cells (**a**). The results are mean ± SEM of four independent experiments performed in triplicate. *p < 0.05, **p < 0.01 by one-way ANOVA

Given the effects of CHIR99021 treatment in T98G cells in terms of cell viability and metabolic state, we further investigated whether this pharmacological modulation of the circadian clock could serve to improve the standard temozolomide (TMZ) treatment for GBM patients. To this end, T98G cells were synchronized and treated with CHIR99021 (8.6 μM), TMZ (844 μM), or a combination of both drugs at 6 or 18 h after synchronization for 48 h. The IC50 of TMZ was previously determined in our laboratory (data not shown). The MTT assay results show a significant reduction in cell viability (~44%) with the combined treatment at 18 h after DEX synchronization compared with the effect of each drug alone (p < 0.05 for TMZ vs TMZ + CHIR and p < 0.01 for CHIR vs TMZ + CHIR by one-way ANOVA) (Fig. 6 b). By contrast, the combined treatment applied at 6 h after synchronization only showed a significant decrease in cell viability with respect to those cells incubated with CHIR99021 treatment alone (p < 0.01 by one-way ANOVA) (Fig. 6a). These results indicate a significant temporal variation in combined chemotherapy treatment.

## Discussion

GBM is a highly vascularized and invasive brain tumor with a poor prognosis. Median patient survival after diagnosis is 12–15 months, there are no full curative therapies and less than 5% of patients survive 5 years (reviewed in (Wagner et al. 2021a)). Recent evidence in the field shows that circadian clock function should be considered a novel target to design new therapeutic strategies or improve the current approaches to treat high-grade gliomas (reviewed in Wagner et al. 2021a; Nettnin et al. 2023)) (Slat et al. 2017; Trebucq et al. 2021, 2024). We thus investigated the effects of pharmacological modulation of the circadian clock in terms of cell viability, proliferation, and migration on GBM cultures using the CK1ε/δ inhibitor (PF670462), the GSK-3 inhibitor (CHIR99021), and the CRY protein stabilizer (KL001). Next, we selected the CHIR99021 inhibitor to evaluate cell cycle distribution, clock protein expression, metabolic parameters, and combined treatment with TMZ. In parallel, we examined the effects of circadian disruption on migration and cell cycle progression in a *Per*2 knockdown GBM model. Dose–response curves using the selective inhibitors of CK1ε/δ, GSK-3, and CRY degradation showed IC50 in the low micromolar range after 48 h of treatment. Similar results in cell viability were observed in another glioma cell line (GL26) after CHIR99021 treatment. The selective pharmacological inhibition of GSK-3 by CHIR99021 has also been shown to impair cell proliferation in epithelioid sarcoma (Russi et al. 2021) and non-small cell lung carcinoma (O’flaherty et al. 2019). Although different GSK-3 inhibitors have been evaluated in GBM models (Kotliarova et al. 2008; Korur et al. 2009; Miyashita et al. 2009; Furuta et al. 2017; Acikgoz et al. 2019; Kitabayashi et al. 2019; Brüning‐richardson et al. 2021), our results demonstrate for the first time that CHIR99021 treatment impairs GBM cell viability. The migratory capacity of tumor cells is a characteristic of more aggressive or malignant cancer types. To this end, we performed wound healing experiments, a well-established technique to determine collective migration that regulates wound repair, cancer invasion, metastasis, immune response, and angiogenesis (Grada et al. 2017). The results revealed that CHIR99021-treated cells (8.6 μM) filled only 35% of the gap after 24 h of treatment compared to 65.1% of wound closure in control cultures. Lower levels of wound closure were also observed when T98G cells were treated with the synthetic agonist of the nuclear receptors REV-ERB. By contrast, no significant differences were observed in the percentage of wound closure between cultures treated with PF670462 (1.5 μM) or KL001 (25.4 μM) and control cells. In this connection, Lin and collaborators showed that KL001 (12 μM) or PF670462 (1.5 μM) treatment for 24 h did not significantly affect cancer cell migration compared to osteosarcoma non-treated cells (Lin et al. 2020). Reports in the literature support our results, suggesting that GSK-3 has a key role in regulating epithelial-mesenchymal transition (EMT) in prostate and bladder tumors (reviewed in (Sahin et al. 2019)). Moreover, the ATP-competitive GSK-3 inhibitor 9-ING-41 impairs the tumorigenicity of renal cancer cells by changing the mesenchymal state to the epithelial direction (Pal et al. 2014). EMT induces numerous biochemical changes in epithelial cells to switch to a mesenchymal phenotype, resulting in an enhanced invasive capacity (Kalluri and Weinberg 2009). Evidence from the laboratory of Richardson and colleagues showed that transcriptional targets of GSK-3β regulate GBM invasion (Bruning-Richardson et al. 2018); Nowicki et al*.* further showed that the effect on migration after GSK-3β inhibition occurred at earlier time points than the effects on cell viability (Nowicki et al. 2008). The type III intermediate filament, vimentin, is a key biomarker of EMT which is normally expressed in mesenchymal cells but is upregulated during cancer metastasis (reviewed in (Usman et al. 2021)). In agreement with this, we reported lower levels of vimentin by western blot after CHIR99021 treatment of T98G cells, highlighting the effect of GSK-3 inhibition in tumor migration.

Since a higher expression and activity of GSK-3β in GBM cultures compared to normal brain samples has been reported (Miyashita et al. 2009) and we described significant effects on cell viability and migration in CHIR99021-treated cells, we decided to examine the cell cycle distribution and metabolic alterations after GSK-3 inhibition in more detail. Uncontrolled proliferation is a hallmark of cancer and GSK-3 has an important role in cell cycle regulation since cyclins are phosphorylated by this kinase (Takahashi-Yanaga and Sasaguri 2007; Mirlashari et al. 2012). We therefore treated T98G cells with CHIR9009 and stained them with propidium iodide followed by flow cytometry analysis. Our results showed a significant alteration in cell cycle distribution, with an increase in the percentage of cells in the S-phase after GSK-3 inhibition compared to control cells. Histograms showing DNA content also revealed ~6% and ~1% of cells in the sub-G_0_ phase in the treated condition and control cultures, respectively. These results suggest a higher percentage of dead cells after CHIR99021 treatment, with the increase in the S-phase possibly indicating cell cycle arrest at this phase. In line with this, various studies have reported alterations in cell cycle distribution after GSK-3 inhibition in GBM cell lines and patient-derived GBM cultures (Acikgoz et al. 2019; Brüning‐richardson et al. 2021). Regarding circadian disruption, propidium iodide-stained cells revealed accumulation in the S-phase in the *Per*2 KD condition after serum stimulation with respect to control cultures. However, no differences were observed between the experimental conditions examined in the count of positive propidium iodide cells. Nevertheless, *Per*2 KD cells also display a significant decrease in wound healing closure as compared to WT cells, strongly indicating that disruption of this clock gene alters the migration, and ultimately the metastatic capacity of these glioma cells.

Regarding clock protein expression after GSK-3 inhibition by CHIR99021 treatment, we observed higher levels of BMAL1 protein by western blot and immunocytochemistry techniques. By contrast, GSK-3 inhibition showed higher levels of the nuclear receptor REV-ERB in the nucleus compared to the control condition. Considering that BMAL1 and REV-ERB proteins are critical regulators of cellular metabolism (reviewed in (Guido et al. 2022)), we evaluated the redox state and lipid droplet (LD) content in CHIR99021-treated cells. Our results showed higher levels of ROS and proportion of cell death (propidium iodide-positive cells) in cultures after treatment with the inhibitor compared to control cells. In coincidence a significant increase in the percentage of cells in the sub-G0 and S phases were found, likely indicating dead cells and cell cycle arrest, respectively. Strikingly, ROS are considered byproducts of normal cellular oxidative processes and have been implicated at the beginning of apoptotic signaling (Tan et al. 1998). In agreement with this, microphotographs of the BMAL1 and REV-ERB immunocytochemistry suggest that chromatin begins to condense, a characteristic event in the mechanism of action of apoptotic cell death. Also, our results clearly show metabolic alteration in GBM cells regarding LD parameters after GSK-3 inhibition. These dynamic organelles are surrounded by a monolayer of polar and amphipathic phospholipids with structural proteins such as the perilipin family (Walther and Farese 2012) and function mainly as regulators of storage and hydrolysis of neutral lipids, which are located in the core of the organelles (Walther and Farese 2012; Yu and Li 2017). LD biology has attracted growing interest in recent years since these organelles are implicated in a plethora of functions such as lipid metabolism, control of protein homeostasis, sequestration of toxic lipid metabolic intermediates, protection from stresses, proliferation of tumors, and platforms for pathogen multiplication and defense (reviewed in (Lundquist et al. 2020)). Our results from Bodipy staining and confocal microscopy reveal a larger average size, number, and percentage area of LDs in CHIR99021-treated cultures with respect to control cells at specific times after synchronization, probably highlighting regulation by the circadian clock. Our findings may reflect either a protective mechanism to detoxify the increase in ROS levels or a metabolic reprogramming mechanism that requires further investigation.

The standard treatment for GBM patients involves the alkylating agent TMZ which methylates DNA at the O^6^-guanine residue site and eventually triggers apoptosis. The methylguanine methyltransferase (MGMT) is a DNA repair enzyme that removes the O^6^-methylguanine and confers resistance to TMZ. The methylation of the MGMT enzyme is considered a biomarker in GBM patients since a subset of them expresses methylated MGMT resulting in an inactive enzyme that prolongs patient survival (Zhang et al. 2012). Mouse models and clinical studies show that different GSK-3 inhibitors improved the monotherapy of TMZ (Furuta et al. 2017; Kitabayashi et al. 2019), probably by silencing MGMT expression via c-Myc-mediated promoter methylation (Pyko et al. 2013). In line with this, we show that the combination of CHIR99021 and TMZ improved the therapeutic strategy compared to the treatment with each drug separately. Our findings underline the importance of designing therapeutic schedules based on the circadian clockwork since the combined treatment was more effective when drugs were applied 18 h after synchronization. In support of these findings, we previously demonstrated temporal susceptibility to treatment with the proteasome inhibitor Bortezomib in both in vitro and in vivo models with a temporal window of higher efficacy between 12 and 24 h after culture synchronization or when chemotherapy was applied at night phase, respectively (Wagner et al. 2018, 2021c). Differential responses to TMZ treatment were also reported by Slat et al*.*, suggesting a *Bmal*1-dependent mechanism (Slat et al. 2017). Survival or tumor weight differences were also observed in tumor-bearing mice according to the drug administration time (Trebucq et al. 2021; Gonzalez-Aponte et al. 2023).

Overall, the pharmacological inhibitors PF670462 and KL001 affect T98G cell viability but do not alter the cellular ability to close the wound. On the other hand, the inhibition of GSK-3 using the ATP-competitive CHIR99021 inhibitor affects GBM viability, migration, and cell cycle distribution as well as clock protein levels and metabolic parameters. Genetic disruption of the clock gene *Per*2 also alters the circadian clock function in migration and cell cycle progression. These results strongly support the idea that the pharmacological modulation and the genetic disruption of the circadian clock severely alter GBM cell biology. Although further studies are needed to elucidate the mechanisms involved, our approach could offer a potential therapeutic strategy by using CHIR99021 combined with TMZ to treat GBM. A significant temporal variation in drug susceptibility was observed in the combined chemotherapy treatment, highlighting the role of the circadian clock at specific times in maximizing therapeutic benefits while minimizing side effects. Based on the literature and our current findings, such a combination will lead to a better understanding of the circadian clock function in health and disease and the design of strategies to maximize therapy effectiveness with lower side effects. Since metabolic reprogramming is a hallmark of cancer cells, the metabolic alterations reported in this manuscript in combination with the circadian modulation could result in a novel therapeutic approach to improve GBM treatment.

## Materials and Methods

### Cell Cultures and Synchronization Protocol

T98G cells are derived from a human GBM (ATCC, Cat. No. CRl-1690, RRUD: CVCL0556) and were tested negative for mycoplasma contamination. The GL26 cell line is derived from a murine GBM and was gently donated by Dr. Marianela Candolfi (Instituto de Investigaciones Biomédicas, Facultad de Medicina, Universidad de Buenos Aires, Argentina). The cell line NIH3T3 (murine fibroblasts, ATCC Cat# CRL-6442, RRID: CVCL_0594) was used as non-cancerous control in cell viability experiments. Cell cultures were grown in Dulbecco’s modified Eagles medium (DMEM) (Gibco, BRL, Invitrogen) supplemented with 10% fetal bovine serum (FBS) at 37ºC and 5% CO_2_ according to (Wagner et al. 2018)**.** Cells were incubated with dexamethasone (DEX, 100 nM) for 1 h at 37ºC to synchronize cell cultures. Then, cells were washed with phosphate-buffered saline (PBS) and collected at different times post-synchronization according to subsequent experiments.

### *Per*2 Disruption by CRISPR/Cas9 Genetic Edition

The human *Per*2 gene was disrupted in T98G cells (ATCC, Cat. No. CRl-1690, RRUD: CVCL0556) using the CRISPR/Cas9 genetic editing tool as previously described (Ran et al. 2013; Wagner et al. 2018). Briefly, we designed single guide RNAs to target the exon 1 of the human *Per*2 gene and subcloned it into the PX459 vector (Addgene) to obtain the PX459-*Per*2 plasmid. The primer sequence corresponding to the single guide RNA was 5′ TCCTCGGCTTGAAACGGCGC 3′. T98G wild-type (WT) cells were transfected with Lipofectamine 2000 (Invitrogen) and selected with puromycin (10 μg/mL) for 4 days. PER2 expression on the isolated clone after serial dilution was evaluated by western blot showing to be at least 50% lower than WT cultures. The complete silencing/knockout of the target gene is not feasible in T98G cells since they have a hyperpentaploid chromosome count (Fig Suppl. 2a, b).

### Pharmacological Treatment and Determination of Cell Viability by Alamarblue Assay

T98G cells were plated in 96-well plates at a density of 3*10^3^ and were allowed to attach overnight at 37ºC. Cultured cells were incubated with PF670462 (CK1ε/δ inhibitor, Santa Cruz), CHIR99021 (GSK-3 Inhibitor XVI, Santa Cruz), or KL001 (Cryptochrome protein stabilizer, Santa Cruz) at different concentrations (0.01, 0.1, 1, 5, 10, 50, and 100 μM) for 48 h at 37 °C. PF670462, CHIR99021, and KL001 stock solutions were resuspended in DMSO to a final concentration of 100 mM, 20 mM, and 10 mM, respectively, according to the manufacturer’s instructions. As control cells, cultures were incubated with the percentage of DMSO corresponding to the maximum concentration of tested drugs. The maximum volume of DMSO in cell culture was always < 1%. After incubation, the culture medium was removed and alamarBlue® 10% v/v dissolved in DMEM supplemented with 10% FBS was added to culture cells for 2–3 h. Then, the fluorescence was measured at a wavelength of 590 nm in a BioteK microplate reader. The IC50 value was calculated according to the equation “log(inhibitor) vs response—Variable slope (four parameters) of the GraphPad Prism software (RRID: SCR_002798). The goodness of fit was evaluated through the R^2^ value.

### Wound Healing Assay

T98G cells were seeded in a 24-well plate at a density of 1*10^5^ cells and incubated overnight at 37ºC to create a monolayer of cells. A P200 pipette tip was used to make a scratch in the middle of the well (t_0_) and the cells were washed with PBS to remove the debris. Fresh culture medium (DMEM + 5% FBS) was added with DMSO (vehicle), PF670462 (1.5 μM), CHIR99021 (8.6 μM), KL001 (25.4 μM), or SR9009 (20 μM) and the cells were incubated for 24 h (t_24_). Similar procedures were used with control cells and *Per*2 knockdown T98G cells without the addition of drugs. Images were taken at t_0_ and t_24_ using the Leica DMI 8 microscope and the wound area was measured using the ImageJ Software (RRID:SCR_003070). The following formula estimated the percentage of wound closure: % = [1 − (wound area at t_24_/wound area at t_0_)] *100%, according to (Grada et al. 2017).

### Immunocytochemistry

Culture cells were seeded in coverslips at a density of 1*10^4^ cells and allowed to attach overnight at 37ºC. T98G cells were treated with DMSO (vehicle) or CHIR99021 (8.6 μM) for 24 h. Then, cells were fixed in 4% paraformaldehyde for 15 min and 10 min in methanol according to (Wagner et al. 2019). Coverslips were washed with PBS and incubated with blocking buffer (PBS supplemented with 0.1% bovine serum albumin, 0.1% Tween 20, and 0.1% glycine) for 1 h at room temperature. Primary antibodies (Table 1) were incubated overnight at 4 °C, and coverslips were washed 3 times with PBS. Finally, goat anti-rabbit immunoglobulin G (IgG; Jackson 549 antibody 1:1000) or goat anti-mouse IgG (Jackson 488 antibody 1:1000) were incubated for 1 h at room temperature. Images were visualized by confocal microscopy (FV1200; Olympus) and analyzed with ImageJ software (RRID:SCR_003070). Cellular nuclei were visualized by DAPI staining.

**Table 1:** Summary of antibodies used, indicating name, host, catalog number, and dilution for immunocytochemistry (ICC) and western blot (WB)

Antibody	Host	Catalog	Dilution	References
REV-ERBα	Mouse	Santa Cruz Biotechnology sc-100910 (RS-14). RRID:AB_2154647	1:100 (ICC) 1:200 (WB)	Wagner et al. (2019)
BMAL1	Rabbit	NOVUS, nb-100–2288. RRID:AB_10000794	1:200 (ICC) 1:500 (WB)	Izumo et al. (2014); Monjes et al. (2022)
α-TUB	Mouse	Merck Millipore, DM1A	1:500 (WB)	
Vimentin	Mouse	Sigma-Aldrich V5255. RID:AB_477625	1:500 (WB)	Huo et al. (2016); Wagner et al. (2019)
PER2	Rabbit	Abcam ab180655. RRID:AB_2630357	1:100 (WB)	Chen et al. (2021); Monjes et al. (2022)
PER1	Rabbit	Abcam. ab3443. RRID:AB_303805	1:100 (ICC)	Wagner et al. (2018); Sun et al. (2020); Monjes et al. (2022)

All antibodies were previously used in WB or ICC

### Western Blot

T98G cells treated with DMSO (vehicle) or CHIR99021 (8.6 μM) for 24 h were harvested in radioimmunoprecipitation assay (RIPA) buffer containing protease inhibitor (Sigma). Proteins were separated by 12% SDS-PAGE according to (Wagner et al. 2018). Primary antibodies described in Table 1 were incubated overnight at 4ºC. Then, membranes were washed with PBS-Tween 0.1% and secondary antibodies were incubated for 1 h at room temperature. Finally, Odyssey IR Imager (LI-COR Biosciences) was used to scan the membranes. The densitometry quantification was performed with ImageJ software (RRID:SCR_003070).

### Cell Cycle Distribution Analysis

T98G cells were incubated with DMSO (vehicle) or CHIR99021 (8.6 μM) in DMEM + 5% FBS for 24 h. Then, cells were collected by trypsinization, washed with PBS, and fixed with ethanol 70% at -20ºC for at least 24 h. Cells were washed with PBS and pellets were resuspended in PBS containing 50 μg/mL propidium iodide and 10 μg/μl RNAse A as reported by (Wagner et al. 2019). Cell cycle distribution was analyzed by flow cytometry and subsequent analysis by FlowJo software (RRID:SCR_008520). For *Per*2 knockdown experiments, control or *Per*2-disrupted cells were arrested in serum-free DMEM for 48 h. Then, the medium was removed and cultures were stimulated with 20% FSB for 20 h (Wagner et al. 2019). Cell cycle analysis was performed as described above.

### Reactive Oxygen Species Determination

T98G cells were treated with DMSO (vehicle) or CHIR99021 (8.6 μM) for 24 h at 37 °C. Then, cells were collected by trypsinization to evaluate the redox state according to (Wagner et al. 2018). Briefly, collected cells were washed with PBS and incubated with 2′,7′- dichlorodihydrofluorescein diacetate (Sigma) at 2 μM final concentration for 30 min at 37 °C protected from light. Cells were washed with PBS and the fluorescence intensity was measured at 530 nm when the sample was excited at 485 nm by flow cytometry. The analysis program used was FlowJo software (RRID:SCR_008520) and live cells were discriminated by propidium iodide stain.

### Lipid Droplet Determination

1*10^4^ cells were seeded in coverslips and synchronized with 100 nM DEX for 1 h at 37 °C. Culture cells were maintained with DMEM supplemented with 5% SFB. After the DEX pulse (t_0_), cells were collected at 6 and 18 h after synchronization. For lipid droplet (LD) staining, cells were fixed with 4% paraformaldehyde for 15 min and washed twice with PBS according to (Monjes et al. 2022). Then, coverslips were incubated with Bodipy (Sigma cat#790389, maximum excitation/emission wavelength: 493/503 nm) at a final concentration of 2 μM for 30 min protected from light. Coverslips were washed twice with PBS and visualized by confocal microscopy at 60× objective (FV1200, Olympus). Cellular nuclei were visualized by DAPI staining. ImageJ software performed the LD average size, percentage area per cell, and number per cell quantification. For LD determination on CHIR-treated cells, cultures were incubated with CHIR99021 (8.6 μM) for 24 h before synchronization and then analyzed as described above.

### Combined Chemotherapeutic Treatments

3*10^3^ T98G cells seeded in 96-well plates were synchronized with 100 nM DEX for 1 h at 37 °C and maintained with DMEM supplemented with 5% SFB. After DEX pulse (t_0_), cells were treated at 6 or 18 h after synchronization with the combination of temozolomide (TMZ, 844 μM) and CHIR99021 (8.6 μM) or each drug alone. After 48 h, cell viability was analyzed by MTT assay as described in (Wagner et al. 2018). T98G cells incubated with DMSO (vehicle) at 6 or 18 h after synchronization were considered 100% viability.

## Supplementary Information

Below is the link to the electronic supplementary material.

Supplementary file1 (DOCX 181 KB)

## Data Availability

The authors declare that the data supporting the findings of this study are available within the paper and its Supplementary Information files. Should any raw data files be needed in another format they are available from the corresponding author upon reasonable request.

## References

[CR1] Acikgoz E, Güler G, Camlar M et al (2019) Glycogen synthase kinase-3 inhibition in glioblastoma multiforme cells induces apoptosis, cell cycle arrest and changing biomolecular structure. Spectrochim Acta Part A Mol Biomol Spectrosc 209:150–164. 10.1016/J.SAA.2018.10.036 10.1016/j.saa.2018.10.036 30388586

[CR2] Badura L, Swanson T, Adamowicz W et al (2007) An inhibitor of casein kinase Iϵ induces phase delays in circadian rhythms under free-running and entrained conditions. J Pharmacol Exp Ther 322:730–738. 10.1124/JPET.107.122846 17502429 10.1124/jpet.107.122846

[CR3] Bass J, Takahashi JS (2010) Circadian integration of metabolism and energetics. Science 330:1349–1354. 10.1126/science.1195027 21127246 10.1126/science.1195027 PMC3756146

[CR4] Beller M, Thiel K, Thul PJ, Jäckle H (2010) Lipid droplets: a dynamic organelle moves into focus. FEBS Lett 584:2176–2182. 10.1016/j.febslet.2010.03.022 20303960 10.1016/j.febslet.2010.03.022

[CR5] Besing RC, Paul JR, Hablitz LM et al (2015) Circadian rhythmicity of active GSK3 isoforms modulates molecular clock gene rhythms in the suprachiasmatic nucleus. J Biol Rhythms 30:155. 10.1177/0748730415573167 25724980 10.1177/0748730415573167 PMC4586074

[CR6] Brasaemle DL, Wolins NE (2012) Packaging of fat: an evolving model of lipid droplet assembly and expansion. J Biol Chem 287:2273–2279. 10.1074/jbc.R111.309088 22090029 10.1074/jbc.R111.309088 PMC3268387

[CR7] Bruning-Richardson DA, Droop DA, Tams DD et al (2018) Identification of transcriptional targets of GSK3 involved in glioblastoma invasion. Neuro Oncol 20:i26. 10.1093/NEUONC/NOX238.117

[CR8] Brüning-richardson A, Shaw GC, Tams D et al (2021) GSK-3 inhibition is cytotoxic in glioma stem cells through centrosome destabilization and enhances the effect of radiotherapy in orthotopic models. Cancers. 10.3390/CANCERS13235939 34885051 10.3390/cancers13235939 PMC8657225

[CR9] Causton HC, Feeney KA, Ziegler CA, O’Neill JS (2015) Metabolic cycles in yeast share features conserved among circadian rhythms. Curr Biol 25:1056–1062. 10.1016/j.cub.2015.02.035 25866393 10.1016/j.cub.2015.02.035 PMC4406945

[CR10] Chen ML, Chen M, Lu D et al (2021) Period 2 regulates CYP2B10 expression and activity in mouse liver. Front Pharmacol. 10.3389/FPHAR.2021.764124/FULL 34887762 10.3389/fphar.2021.764124 PMC8650840

[CR11] Dunlap JC (1999) Molecular bases for circadian clocks. Cell 96:271–290. 10.1016/S0092-8674(00)80566-8 9988221 10.1016/s0092-8674(00)80566-8

[CR87] Dunlap JC, Loros JJ, DeCoursey PJ (eds) (2004) Chronobiology: biological timekeeping. Sinauer Associates: Sunderland, MA, USA, ISBN 087893149X

[CR12] Farese RV, Walther TC (2009) Lipid droplets finally get a little R-E-S-P-E-C-T. Cell 139:855–860. 10.1016/j.cell.2009.11.005 19945371 10.1016/j.cell.2009.11.005 PMC3097139

[CR13] Furuta T, Sabit H, Dong Y et al (2017) Oncotarget 22811 www.impactjournals.com/oncotarget biological basis and clinical study of glycogen synthase kinase-3β-targeted therapy by drug repositioning for glioblastoma. Oncotarget 8:22811–22824 28423558 10.18632/oncotarget.15206 PMC5410264

[CR14] Golombek DA, Rosenstein RE (2010) Physiology of circadian entrainment. Physiol Rev 90:1063–1102. 10.1152/physrev.00009.2009 20664079 10.1152/physrev.00009.2009

[CR15] Gonzalez-Aponte MF, Damato AR, Trebucq LL et al (2023) Circadian regulation of MGMT expression and promoter methylation underlies daily rhythms in TMZ sensitivity in glioblastoma. bioRxiv. 10.1101/2023.09.13.557630 38277015 10.1007/s11060-023-04535-9 PMC11301575

[CR16] Grada A, Otero-Vinas M, Prieto-Castrillo F et al (2017) Research techniques made simple: analysis of collective cell migration using the wound healing assay. J Invest Dermatol 137:e11–e16. 10.1016/J.JID.2016.11.020 28110712 10.1016/j.jid.2016.11.020

[CR17] Guido ME, Garbarino-Pico E, Contin MA et al (2010) Inner retinal circadian clocks and non-visual photoreceptors: novel players in the circadian system. Prog Neurobiol 92:484–504. 10.1016/J.PNEUROBIO.2010.08.005 20736045 10.1016/j.pneurobio.2010.08.005

[CR18] Guido ME, Monjes NM, Wagner PM, Salvador GA (2022) Circadian regulation and clock-controlled mechanisms of glycerophospholipid metabolism from neuronal cells and tissues to fibroblasts. Mol Neurobiol 59:326–353. 10.1007/S12035-021-02595-4 34697790 10.1007/s12035-021-02595-4

[CR19] Hirota T, Lewis WG, Liu AC et al (2008) A chemical biology approach reveals period shortening of the mammalian circadian clock by specific inhibition of GSK-3β. Proc Natl Acad Sci USA 105:20746. 10.1073/PNAS.0811410106 19104043 10.1073/pnas.0811410106 PMC2606900

[CR20] Hirota T, Lee JW, St. John PC et al (2012) Identification of small molecule activators of cryptochrome. Science 337:1094. 10.1126/SCIENCE.1223710 22798407 10.1126/science.1223710 PMC3589997

[CR21] Huo Y, Zheng Z, Chen Y et al (2016) Downregulation of vimentin expression increased drug resistance in ovarian cancer cells. Oncotarget 7:45876–45888. 10.18632/ONCOTARGET.9970 27322682 10.18632/oncotarget.9970 PMC5216767

[CR22] Izumo M, Pejchal M, Schook AC et al (2014) Differential effects of light and feeding on circadian organization of peripheral clocks in a forebrain Bmal1 mutant. Elife. 10.7554/ELIFE.04617 25525750 10.7554/eLife.04617 PMC4298698

[CR23] Kaladchibachi SA, Doble B, Anthopoulos N et al (2007) Glycogen synthase kinase 3, circadian rhythms, and bipolar disorder: a molecular link in the therapeutic action of lithium. J Circadian Rhythms 5:3. 10.1186/1740-3391-5-3 17295926 10.1186/1740-3391-5-3 PMC1803776

[CR24] Kalluri R, Weinberg RA (2009) The basics of epithelial-mesenchymal transition. J Clin Invest 119:1420. 10.1172/JCI39104 19487818 10.1172/JCI39104 PMC2689101

[CR25] Kitabayashi T, Dong Y, Furuta T et al (2019) Identification of GSK3β inhibitor kenpaullone as a temozolomide enhancer against glioblastoma. Sci Rep. 10.1038/S41598-019-46454-8 31296906 10.1038/s41598-019-46454-8 PMC6624278

[CR26] Korur S, Huber RM, Sivasankaran B et al (2009) GSK3β regulates differentiation and growth arrest in glioblastoma. PLoS ONE 4:e7443. 10.1371/JOURNAL.PONE.0007443 19823589 10.1371/journal.pone.0007443 PMC2757722

[CR27] Kotliarova S, Pastorino S, Kovell LC et al (2008) Glycogen synthase kinase 3 inhibition induces glioma cell death through c-MYC, NF-κB and glucose regulation. Cancer Res 68:6643–6651. 10.1158/0008-5472.CAN-08-0850 18701488 10.1158/0008-5472.CAN-08-0850 PMC2585745

[CR28] Kurabayashi N, Hirota T, Sakai M et al (2010) DYRK1A and glycogen synthase kinase 3β, a dual-kinase mechanism directing proteasomal degradation of CRY2 for circadian timekeeping. Mol Cell Biol 30:1757. 10.1128/MCB.01047-09 20123978 10.1128/MCB.01047-09 PMC2838083

[CR29] Li W, Wang Z, Cao J et al (2023) Perfecting the life clock: the journey from PTO to TTFL. Int J Mol Sci. 10.3390/IJMS24032402 36768725 10.3390/ijms24032402 PMC9916482

[CR30] Lin HH, Robertson KL, Bisbee HA, Farkas ME (2020) Oncogenic and circadian effects of small molecules directly andindirectly targeting the core circadian clock. Integr Cancer Ther. 10.1177/1534735420924094 32493076 10.1177/1534735420924094 PMC7273620

[CR31] Lundquist PK, Shivaiah K-K, Espinoza-Corral R (2020) Lipid droplets throughout the evolutionary tree. Prog Lipid Res. 10.1016/j.plipres.2020.101029 32348789 10.1016/j.plipres.2020.101029

[CR32] Ma D, Hou L, Xia H et al (2020) PER2 inhibits proliferation and stemness of glioma stem cells via the Wnt/ß-catenin signaling pathway. Oncol Rep 44:533–542. 10.3892/or.2020.7624 32468039 10.3892/or.2020.7624 PMC7336516

[CR33] Mirlashari MR, Randen I, Kjeldsen-Kragh J (2012) Glycogen synthase kinase-3 (GSK-3) inhibition induces apoptosis in leukemic cells through mitochondria-dependent pathway. Leuk Res 36:499–508. 10.1016/J.LEUKRES.2011.11.013 22177455 10.1016/j.leukres.2011.11.013

[CR34] Miyashita K, Kawakami K, Nakada M et al (2009) Potential therapeutic effect of glycogen synthase kinase 3beta inhibition against human glioblastoma. Clin Cancer Res 15:887–897. 10.1158/1078-0432.CCR-08-0760 19188159 10.1158/1078-0432.CCR-08-0760

[CR35] Monjes NM, Wagner PM, Guido ME (2022) Disruption of the molecular clock severely affects lipid metabolism in a hepatocellular carcinoma cell model. J Biol Chem 298:102551. 10.1016/J.JBC.2022.102551 36183836 10.1016/j.jbc.2022.102551 PMC9637785

[CR36] Nettnin EA, Nguyen T, Arana S et al (2023) Review: therapeutic approaches for circadian modulation of the glioma microenvironment. Front Oncol 13:1295030. 10.3389/FONC.2023.1295030/BIBTEX 38173841 10.3389/fonc.2023.1295030 PMC10762863

[CR37] Nowicki MO, Dmitrieva N, Stein AM et al (2008) Lithium inhibits invasion of glioma cells; possible involvement of glycogen synthase kinase-3. Neuro Oncol 10:690. 10.1215/15228517-2008-041 18715951 10.1215/15228517-2008-041 PMC2666245

[CR38] O’flaherty L, Shnyder SD, Cooper PA et al (2019) Tumor growth suppression using a combination of taxol-based therapy and GSK3 inhibition in non-small cell lung cancer. PLoS ONE. 10.1371/journal.pone.0214610 30969984 10.1371/journal.pone.0214610 PMC6457575

[CR39] Ortega-Campos SM, Verdugo-Sivianes EM, Amiama-Roig A et al (2023) Interactions of circadian clock genes with the hallmarks of cancer. Biochim Biophys Acta - Rev Cancer 1878:188900. 10.1016/J.BBCAN.2023.188900 37105413 10.1016/j.bbcan.2023.188900

[CR40] Pal K, Cao Y, Gaisina IN et al (2014) Inhibition of GSK-3 induces differentiation and impaired glucose metabolism in renal cancer. Mol Cancer Ther 13:285. 10.1158/1535-7163.MCT-13-0681 24327518 10.1158/1535-7163.MCT-13-0681 PMC3956125

[CR41] Putker M, Wong DCS, Seinkmane E et al (2021) CRYPTOCHROMES confer robustness, not rhythmicity, to circadian timekeeping. EMBO J. 10.15252/EMBJ.2020106745 33491228 10.15252/embj.2020106745 PMC8013833

[CR42] Pyko IV, Nakada M, Sabit H et al (2013) Glycogen synthase kinase 3β inhibition sensitizes human glioblastoma cells to temozolomide by affecting O 6 -methylguanine DNA methyltransferase promoter methylation via c-Myc signaling. Carcinogenesis 34:2206–2217. 10.1093/CARCIN/BGT182 23715499 10.1093/carcin/bgt182

[CR43] Qin X, Mori T, Zhang Y, Johnson CH (2015) PER2 differentially regulates clock phosphorylation versus transcription by reciprocal switching of CK1ε activity. J Biol Rhythms 30:206. 10.1177/0748730415582127 25994100 10.1177/0748730415582127 PMC4697459

[CR44] Qiu B, Simon MC (2016) BODIPY 493/503 Staining of Neutral Lipid Droplets for Microscopy and Quantification by Flow Cytometry. Bio-protocol 6:. 10.21769/BIOPROTOC.1912 10.21769/BioProtoc.1912 PMC5448404 28573161

[CR45] Ran FA, Hsu PD, Wright J et al (2013) Genome engineering using the CRISPR-Cas9 system. Nat Protoc 8:2281–2308. 10.1038/nprot.2013.143 24157548 10.1038/nprot.2013.143 PMC3969860

[CR46] Russi S, Sgambato A, Bochicchio AM et al (2021) Chir99021, trough gsk-3β targeting, reduces epithelioid sarcoma cell proliferation by activating mitotic catastrophe and autophagy. Int J Mol Sci. 10.3390/IJMS222011147/S1 34681807 10.3390/ijms222011147 PMC8538073

[CR47] Sahar S, Zocchi L, Kinoshita C et al (2020) Regulation of BMAL1 protein stability and circadian function by GSK3b-mediated phosphorylation. Prog Lipid Res. 10.1371/journal.pone.0008561 10.1371/journal.pone.0008561 PMC2797305 20049328

[CR48] Sahin I, Eturi A, De Souza A et al (2019) Glycogen synthase kinase-3 beta inhibitors as novel cancer treatments and modulators of antitumor immune responses. Cancer Biol Ther. 10.1080/15384047.2019.1595283 30975030 10.1080/15384047.2019.1595283 PMC6606036

[CR49] Slat EA, Sponagel J, Marpegan L et al (2017) Cell-intrinsic, Bmal1-dependent circadian regulation of temozolomide sensitivity in glioblastoma. J Biol Rhythms 32:121–129. 10.1177/0748730417696788 28470120 10.1177/0748730417696788 PMC6410359

[CR50] Spengler ML, Kuropatwinski KK, Schumer M, Antoch MP (2009) A serine cluster mediates BMAL-dependent CLOCK phosphorylation and degradation. Cell Cycle 8:4138. 10.4161/CC.8.24.10273 19946213 10.4161/cc.8.24.10273 PMC4073639

[CR51] Stevens RG, Hansen J, Costa G et al (2011) Considerations of circadian impact for defining “shift work” in cancer studies: IARC Working Group Report. Occup Environ Med 68:154–162. 10.1136/oem.2009.053512 20962033 10.1136/oem.2009.053512

[CR52] Stupp R, Hegi ME, Mason WP et al (2009) Effects of radiotherapy with concomitant and adjuvant temozolomide versus radiotherapy alone on survival in glioblastoma in a randomised phase III study: 5-year analysis of the EORTC-NCIC trial. Lancet Oncol 10:459–466. 10.1016/S1470-2045(09)70025-7 19269895 10.1016/S1470-2045(09)70025-7

[CR53] Sulli G, Lam MTY, Panda S (2019) Interplay between circadian clock and cancer: new frontiers for cancer treatment. Trends Cancer 5:475–494 31421905 10.1016/j.trecan.2019.07.002 PMC7120250

[CR54] Sun Q, Yang Y, Wang Z et al (2020) PER1 interaction with GPX1 regulates metabolic homeostasis under oxidative stress. Redox Biol 37:101694. 10.1016/J.REDOX.2020.101694 32896721 10.1016/j.redox.2020.101694 PMC7484554

[CR55] Takahashi-Yanaga F, Sasaguri T (2007) GSK-3β regulates cyclin D1 expression: a new target for chemotherapy. Cell Signal. 10.1016/j.cellsig.2007.10.018 18023328 10.1016/j.cellsig.2007.10.018

[CR86] Tan S, Sagara Y, Liu Y, Maher P, Schubert D (1998) The regulation of reactive oxygen species production during programmed cell death. J Cell Biol. 141(6):1423–1432. 10.1083/jcb.141.6.1423 9628898 10.1083/jcb.141.6.1423 PMC2132785

[CR56] Thakkar JP, Dolecek TA, Horbinski C et al (2014) Epidemiologic and molecular prognostic review of glioblastoma. Cancer Epidemiol Biomarkers Prev 23:1985–1996 25053711 10.1158/1055-9965.EPI-14-0275 PMC4185005

[CR57] Trebucq LL, Cardama GA, Menna PL et al (2021) Timing of novel drug 1A–116 to circadian rhythms improves therapeutic effects against glioblastoma. Pharmaceutics. 10.3390/PHARMACEUTICS13071091 34371781 10.3390/pharmaceutics13071091 PMC8309043

[CR58] Trebucq LL, Salvatore N, Wagner PM et al (2024) Circadian clock gene bmal1 acts as a tumor suppressor gene in a mice model of human glioblastoma. Mol Neurobiol. 10.1007/S12035-023-03895-7 38180613 10.1007/s12035-023-03895-7

[CR59] Usman S, Waseem NH, Nguyen TKN et al (2021) Vimentin is at the heart of epithelial mesenchymal transition (EMT) mediated metastasis. Cancers 13:4985. 10.3390/CANCERS13194985 34638469 10.3390/cancers13194985 PMC8507690

[CR60] Wagner PM, Sosa Alderete LG, Gorné LD et al (2018) Proliferative glioblastoma cancer cells exhibit persisting temporal control of metabolism and display differential temporal drug susceptibility in chemotherapy. Mol Neurobiol. 10.1007/s12035-018-1152-3 29881948 10.1007/s12035-018-1152-3

[CR61] Wagner PM, Monjes NM, Guido ME (2019) Chemotherapeutic effect of SR9009, a REV-ERB agonist, on the human glioblastoma T98G cells. ASN Neuro 11:175909141989271. 10.1177/1759091419892713 10.1177/1759091419892713 PMC6909277 31825658

[CR62] Wagner PM, Prucca CG, Caputto BL, Guido ME (2021a) Adjusting the molecular clock: the importance of circadian rhythms in the development of glioblastomas and its intervention as a therapeutic strategy. Int J Mol Sci 8289(22):8289. 10.3390/IJMS22158289 10.3390/ijms22158289 PMC8348990 34361055

[CR63] Wagner PM, Prucca CG, Velazquez FN et al (2021b) Temporal regulation of tumor growth in nocturnal mammals: In vivo studies and chemotherapeutical potential. FASEB J 35:e21231. 10.1096/fj.202001753R 33428275 10.1096/fj.202001753R

[CR64] Walther TC, Farese RV (2012) Lipid droplets and cellular lipid metabolism. Annu Rev Biochem 81:687–714. 10.1146/annurev-biochem-061009-102430 22524315 10.1146/annurev-biochem-061009-102430 PMC3767414

[CR65] Wong DC, O’neill JS (2018) Non-transcriptional processes in circadian rhythm generation introduction: the utility of biological timekeeping at multiple levels of organisation. Curr Opin Physiol 5:117–132. 10.1016/j.cophys.2018.10.003 30596188 10.1016/j.cophys.2018.10.003 PMC6302373

[CR66] Yao J, Wen HJ, Jun CY et al (2023) Lycium barbarum glycopeptide targets PER2 to inhibit lipogenesis in glioblastoma by downregulating SREBP1c. Cancer Gene Ther 308(30):1084–1093. 10.1038/s41417-023-00611-4 10.1038/s41417-023-00611-4 PMC10425286 37069338

[CR67] Yu J, Li P (2017) The size matters: regulation of lipid storage by lipid droplet dynamics. Sci China Life Sci 60:46–56. 10.1007/s11427-016-0322-x 27981432 10.1007/s11427-016-0322-x

[CR68] Zhang J, F.G. Stevens M, D. Bradshaw T, (2012) Temozolomide: mechanisms of action, repair and resistance. Curr Mol Pharmacol 5:102–114. 10.2174/1874467211205010102 22122467 10.2174/1874467211205010102

