# Supplementary material for: Pharmacological Modulation of the Cytosolic Oscillator Affects Glioblastoma Cell Biology
Source: Cell Mol Neurobiol. 2024 Jun 22;44:51. doi: 10.1007/s10571-024-01485-2 (PMC11193694; doi:10.1007/s10571-024-01485-2)
Supplement: Supplementary file 1 — Supplementary file1 (DOCX 181 KB) [file 10571_2024_1485_MOESM1_ESM.docx]

**Suppl. Fig. 1: Effect of CHIR99021 treatment on GL26, and NIH 3T3 cell viability.** Dose-response curves of CHIR99021 treatment in GL26 **(a)** and NIH 3T3 **(b)** cells showed an IC50 value of 38.7 μM (R^2^=0.92)**,** and 91.2 μM (R^2^=0.99), respectively after 48 hours of treatment. The results are mean ± SD (n=3/group).





**Suppl. Fig. 2: Genetic disruption of the clock gene *Per*2 by CRISPR/Cas9 tool on T98G cells. a)** PER2 expression was evaluated by western blot in the isolated clone obtained by serial dilution after transfection and antibiotic selection in T98G cultures. Results showed at least 50% lower levels of PER2 in *Per*2 KD cells than in non-transfected cultures. Tubulin (TUB) was used as the loading control**. b)** *Bmal*1 expression was evaluated by RT-PCR in *Per*2 KD and control cultures. Results showed a higher expression of *Bmal*1 after genetic disruption of *Per*2. Tata binding protein (*tbp)* gene was used as housekeeping.





**Suppl. Fig. 3: BMAL1 and vimentin expression levels in T98G cells after CHIR99021 treatment.** Cultures were incubated with CHIR99021 (8.6 μM) for 24 h and protein levels were detected by western blot. **a)** Representative western blot of BMAL1 and vimentin (VIM) in CHIR99021-treated cells and control cultures. Tubulin (TUB) was used as the loading control. **b)** Histograms of relative protein levels indicating an increase in BMAL1 and a decrease in vimentin levels in T98G cells after GSK-3 inhibition (gray bars) as compared to non-treated conditions (black bar). **p < 0.01 by unpaired t-test.
